# Outbreak of plasmid-mediated NDM-1-producing *Klebsiella pneumoniae* ST105 among neonatal patients in Yunnan, China

**DOI:** 10.1186/s12941-016-0124-6

**Published:** 2016-02-19

**Authors:** Rui Zheng, Qian Zhang, Yidan Guo, Yue Feng, Li Liu, Amei Zhang, Yue Zhao, Xiaoyu Yang, Xueshan Xia

**Affiliations:** Faculty of Environmental Science and Engineering, Kunming University of Science and Technology, Kunming, Yunnan China; Faculty of Life Science and Technology, Kunming University of Science and Technology, No. 727 Jing Ming Road, Chenggong District, Kunming, Yunnan China; Department of Clinical Laboratory, First People’s Hospital of Yunnan province, Kunming, Yunnan China; Department of Clinical Laboratory, Kunming City Maternal and Child health Hospital, Kunming, Yunnan China; Yunnan Center for Disease Control and Prevention, Kunming, Yunnan China

**Keywords:** CPE, NDM-1, ST105, China

## Abstract

**Background:**

In the past decade, the carbapenemase-producing *Enterobacteriaceae* (CPE) have been reported worldwide. Emergence of carbapenemase-producing strains among *Enterobacteriaceae* has been a challenge for treatment of clinical infection. The present study was undertaken to investigate the characteristics of carbapenem-resistant *Klebsiella pneumoniae* recovered from an outbreak that affected 17 neonatal patients in neonatal intensive care unit (NICU) of Kunming City Maternal and Child health Hospital, which is located in the Kunming city in far southwest of China.

**Methods:**

Minimum inhibitory concentrations (MICs) for antimicrobial agents were determined according to the guidelines of the Clinical and Laboratory Standards Institute (CLSI); Modified Hodge test and Carba-NP test were preformed to identified the phenotypes of carbapenemases producing; To determine whether carbapenem resistance was transferable, a conjugation experiment was carried out in mixed broth cultures; Resistant genes were detected by using PCR and sequencing; Plasmids were typed by PCR-based replicon typing method; Clone relationships were analyzed by using multilocus-sequence typing (MLST) and pulsed field gel electrophoresis (PFGE).

**Results:**

Eighteen highly carbapenem-resistant *Klebsiella pneumoniae* were isolated from patients in NICU and one carbapenem-resistant *K. pneumoniae* isolate was detected in incubator water. All these isolates harbored *bla*_NDM-1_. Moreover, other resistance genes, viz., *bla*_IMP-4_*, bla*_SHV-1_*, bla*_TEM-1_*, bla*_CTX-M-15_*, qnrS1, qnrB4*, and *aacA4* were detected. The *bla*_NDM-1_ gene was located on a ca. 50 kb IncFI type plasmid. PFGE analysis showed that NDM-1-producing *K. pneumoniae* were clonally related and MLST assigned them to sequence type 105.

**Conclusions:**

NDM-1 producing strains present in the hospital environment pose a potential risk and the incubator water may act as a diffusion reservoir of NDM-1- producing bacteria. Nosocomial surveillance system should play a more important role in the infection control to limit the spread of these pathogens.

## Background

Gram-negative bacilli are the most important cause of healthcare associated infections [1]. Among these, *Enterobacteriaceae* continue to be an important cause of such infections [2], particularly the carbapenemase-producing *Enterobacteriaceae* (CPE) in developing countries, such as China [3]. In the past decade, the CPEs have been reported worldwide, including KPC-, GES-, VIM-, IMP-, GIM-, NDM- and OXA- types [4]. Among them, the NDM-type carbapenemase is a novel metalo-beta-lactamase that was identified for the first time in 2008 [5]. NDM-1 carbapenemase belongs to class B of Ambler β-lactamases, and efficiently hydrolyses a broad range of β-lactams, including penicillins, cephalosporins, and carbapenems, except for aztreonam. Up to this day, the emergence of carbapenemase-producing strains among *Enterobacteriaceae* has been a challenge for treatment of clinical infection [6].

Plasmid-mediated drug resistance is one of the most serious problems in the treatment of infectious diseases due to the horizontal transfer of plasmids account for the dissemination of resistance genes and the emergence of drug resistant strains [7, 8]. Carbapenemase-producing strains are most often associated with many non-β-lactam-resistance genes, because of their locations on plasmids [9], which made therapeutic options for infections were very limited.

*Klebsiella pneumoniae* was a leading cause of nosocomial infections and spread rapidly in health care settings due to efficiency of colonization and rapid development of resistance to a wide range of antimicrobials [10]. Recently, *K. pneumoniae* harboring *bla*_NDM-1_ were emergencing in China, which should pay great attention [11, 12]. Therefore, investigation of the molecular characteristics of NDM-1-producing *K. pneumoniae* is critical. Here, we identified 19 *K. pneumoniae* harboring *bla*_NDM-1_, the transmission of these NDM-1-producing *K. pneumoniae* among neonatal patients at Kunming City Maternal and Child health Hospital was delineated in this study.

## Methods

### Bacterial isolates

Kunming City Maternal and Child health Hospital was a 200-bed tertiary care community health facility in the provincial capital, Kunming City. Carbapenem-resistant *Enterobacteriaceae* (CRE) isolates were rare in this facility prior to this outbreak. On January 22, 2014, one *K. pneumoniae* strain (M1) was isolated from a sputum specimen, obtained from a neonatal patient in the neonatal intensive care unit (NICU), this strain was resistant to carbapenems including imipenem and meropenem. On January 23, 2014, another carbapenem-resistant *K. pneumoniae* strain (M2) was isolated from a stool sample obtained from another neonate in the same ward. We screened the rectal swab samples taken from patients in the NICU ward; simultaneously, environmental swabs of bed linen, stethoscopes, doorknobs, and water in the neonatal incubator, and the hand swabs obtained from doctors and nurses, were also collected. All swabs were inoculated on the Mueller–Hinton plates containing 2 μg/mL meropenem. The colonies that grew on the selection medium and clinical isolates with decreased susceptibility to carbapenems were picked and identified using a VITEK 2 Compact (bioMérieux, Marcy l’Etoile, France).

### Detection of phenotypes

The production of carbapenemases was evaluated in all isolates using a Modified Hodge test [13] and Carba NP test [14], as previously described.

### Antimicrobial susceptibility testing

MICs for antimicrobial agents were determined by using the microdilution susceptibility testing method, according to the guidelines of the CLSI [15]. The antibiotics tested included imipenem, meropenem, ceftazidime, aztreonam, piperacillin, piperacillin/tazobactam, tigecycline, levofloxacin, and amikacin. MIC results were interpreted as specified by CLSI [13], except for tigecycline, which was interpreted as defined by the US Food and Drug Administration (susceptible: MIC ≤ 2 mg/L; resistant: MIC ≥ 8 mg/L). *Escherichia coli* ATCC 25922 was used as quality control.

### Detection of drug-resistant genes

Bacterial chromosomal DNA was obtained from clinical strains and transconjugants with a TIANamp Bacterial DNA Kit according to the manufacturer’s instructions (TIANGEN BIOTECH, Beijing, China). PCR and DNA sequence analysis were performed to confirm the presence of drug-resistant genes. The primers used in this study were described previously [3, 16]. β-lactamase genes, including, Ambler class A (*bla*_CTX-M_*, bla*_TEM_*, bla*_SHV_*, bla*_KPC_*, bla*_IMI_ and *bla*_GES_), class B (*bla*_VIM_*, bla*_IMP_*, bla*_NDM_, and *bla*_SPM_), class C (*bla*_CMY_*, bla*_ACT-1_, and *bla*_DHA-1_), and class D (*bla*_OXA-48_) were detected in all clinical isolates and their transconjugants. Moreover, genes related to quinolone activity including *qnrA, qnrB,* and *qnrS*, *integron* genes and the *aac* gene were also detected. Products were sequenced on an ABI PRISM 3730AXL sequencer analyzer and compared with the reported sequences from GenBank.

### Molecular typing

NDM-1-producing strains were genotyped by using MLST and PFGE. Seven housekeeping genes (*gapA, infB, mdh, pgi, phoE, rpoB,* and *tonB*) were amplified according to the protocol described on the MLST website [17]. PFGE were performed according to the procedure described by Pulse Net from the website of the Centers for Disease Control and Prevention [18]. *Salmonella enterica* serotype H9812 was used as a marker. The XbaI restriction patterns were analyzed and interpreted according to the criteria of Tenover et al. [19].

### Analysis of plasmid and conjugation experiment

In order to determine whether carbapenem resistance was transferable, a conjugation experiment was carried out in mixed broth cultures. *Escherchia coli* J53 (Az^R^) was used as the recipient strain. Test strains and the recipient strain were grown separately overnight in Luria–Bertani broth at 35 °C with shaking. Cultures (2 ml) of test strains and recipient strains were mixed in a tube and incubated at 35 °C for 4 h with shaking. Then, 50 μL of the mixture was placed on Mueller–Hinton agar containing 2 μg/mL meropenem and 200 mg/L sodium azide and incubated at 35 °C for 20 h. The colonies that grew on this medium were regarded as the products of successful conjugation and were picked up and identified using a VITEK 2 Compact. Plasmid DNA from donors and transformants were extracted with a TIANprep Plasmid Maxi Plasmid Kit according to the manufacturer’s instructions (TIANGEN BIOTECH, Beijing, China) and was electrophoresed on 0.8 % agarose gels at 100 V for 4 h. The plasmid replicons of the *bla*_NDM-1_-encoding plasmids were typed by using the PCR-based replicon typing method described previously [20].

## Results

### Bacterial isolates

Eighteen carbapenem-resistant *K. pneumoniae* strains (M1–M18) were isolated from 17 patients in a variety specimens including sputum, stool, and blood; and one carbapenem-resistant *K. pneumoniae* (M19) was detected in incubator water. The CRE outbreak was declared on March 31, 2014. Resistace screening was performed for all patients in the NICU until no further transmission was detected. All the 17 patients had received meropenem treatment as initial monotherapy, one patient died on February 10, 2014, while all others were recovered.

### Phenotypes and drug-resistant genes

All strains (M1–M19) harbored *bla*_NDM-1_, a carbapenemase-encoding gene. M3, M5, M8, M9, M17, M18, and M19 co-harbor another carbapenemase gene *bla*_IMP-4_. Thirteen of 19 isolates showed positive phenotypic screening results for the Modified Hodge test, the positivity rate was 72 %, while the positive rate for the Carba-NP test was 100 %. Other β-lactamase genes were identified in 19 strains, including those of the *bla*_TEM_, *bla*_CTX-M_, and *bla*_SHV_. The *qnr* and *aac* genes were also detected; no AmpC-like enzymes and *integron* genes were found. The remaining resistance genes that were evaluated were not detected. Details on these findings are shown in Table 1.

**Table 1:** Drug resistance profiles and resistance mechanisms of NDM-1-producing *K. pneumoniae*

Strains	Organism	Source	*NDM*	*IMP*	*CTX*	*SHV*	*TEM*	*QnrB*	*qnrS*	*aac*	MICs, μg/mL									
											MEM	IPM	CAZ	FOX	AMK	TIG	PIP	PIP/TZB	ATM	LEV
M1	KPN	Sputum	*NDM*-*1*	–	*CTX*-*M*-*15*	*SHV*-*1*	*TEM*-*1*	–	–	–	128	32	>128	>128	1	0.25	>128	>128/4	>128	<0.06
M2	KPN	Stool	*NDM*-*1*	–	*CTX*-*M*-*15*	*SHV*-*1*	–	*qnrB4*	–	*aacA4*	64	32	>128	>128	2	0.25	>128	>128/4	4	0.25
M3	KPN	Stool	*NDM*-*1*	*IMP*-*4*	*CTX*-*M*-*15*	*SHV*-*1*	–	–	*qnrS1*	*aacA4*	64	16	>128	>128	1	0.25	>128	>128/4	8	<0.06
M4	KPN	Stool	*NDM*-*1*	–	*CTX*-*M*-*15*	*SHV*-*1*	–	*qnrB4*	*qnrS1*	*aacA4*	128	32	>128	>128	2	0.25	>128	>128/4	4	<0.06
M5	KPN	Stool	*NDM*-*1*	*IMP*-*4*	*CTX*-*M*-*15*	*SHV*-*1*	–	*qnrB4*	*qnrS1*	*aacA4*	128	16	>128	>128	1	0.25	>128	>128/4	32	<0.06
M6	KPN	Stool	*NDM*-*1*	–	*CTX*-*M*-*15*	*SHV*-*1*	–	–	–	–	64	16	>128	>128	2	0.25	>128	>128/4	4	<0.06
M7	KPN	Blood	*NDM*-*1*	–	*CTX*-*M*-*15*	*SHV*-*1*	–	–	–	–	64	16	>128	>128	1	0.25	>128	>128/4	8	0.25
M8	KPN	Blood	*NDM*-*1*	*IMP*-*4*	*CTX*-*M*-*15*	*SHV*-*1*	–	–	*qnrS1*	–	64	16	>128	>128	1	0.25	>128	>128/4	16	<0.06
M9	KPN	Sputum	*NDM*-*1*	*IMP*-*4*	*CTX*-*M*-*15*	*SHV*-*1*	–	–	*qnrS1*	*aacA4*	64	32	>128	>128	1	0.25	>128	>128/4	8	<0.06
M10	KPN	Stool	*NDM*-*1*	–	*CTX*-*M*-*15*	*SHV*-*1*	–	–	–	–	64	32	>128	>128	1	0.25	>128	>128/4	4	<0.06
M11	KPN	Stool	*NDM*-*1*	–	*CTX*-*M*-*15*	*SHV*-*1*	–	–	*qnrS1*	*aacA4*	32	4	>128	>128	1	0.5	>128	>128/4	4	<0.06
M12	KPN	Stool	*NDM*-*1*	–	*CTX*-*M*-*15*	*SHV*-*1*	–	*qnrB4*	*qnrS1*	–	64	16	>128	>128	1	0.5	>128	>128/4	64	<0.06
M13	KPN	Stool	*NDM*-*1*	–	*CTX*-*M*-*15*	*SHV*-*1*	–	*qnrB4*	*qnrS1*	–	64	128	>128	>128	1	1	>128	>128/4	32	<0.06
M14	KPN	Blood	*NDM*-*1*	–	*CTX*-*M*-*15*	*SHV*-*1*	–	*qnrB4*	–	–	64	32	>128	>128	1	0.25	>128	>128/4	16	<0.06
M15	KPN	Blood	*NDM*-*1*	–	*CTX*-*M*-*15*	*SHV*-*1*	–	*qnrB4*	–	–	64	32	>128	>128	1	0.25	>128	>128/4	4	<0.06
M16	KPN	Stool	*NDM*-*1*	–	*CTX*-*M*-*15*	*SHV*-*1*	–	*qnrB4*	–	*aacA4*	64	>128	>128	>128	8	0.5	>128	>128/4	16	8
M17	KPN	Sputum	*NDM*-*1*	*IMP*-*4*	*CTX*-*M*-*15*	*SHV*-*1*	–	*qnrB4*	*qnrS1*	*aacA4*	128	16	>128	>128	1	0.5	>128	>128/4	32	<0.06
M18	KPN	Sputum	*NDM*-*1*	*IMP*-*4*	*CTX*-*M*-*15*	*SHV*-*1*	–	*qnrB4*	*qnrS1*	*aacA4*	64	16	>128	>128	1	0.5	>128	>128/4	32	<0.06
M19	KPN	iw	*NDM*-*1*	*IMP*-*4*	*CTX*-*M*-*15*	*SHV*-*1*	–	*qnrB4*	*qnrS1*	*aacA4*	64	32	>128	>128	1	0.5	>128	>128/4	16	<0.06

*KPN* *Klebsiella pneumoniae*, *ECO* *Escherichia coli*, *MEM* meropenem, *IPM* imipenem, *CAZ* ceftazidime, *FOX* cefoxitin, *AMK* amikacin, *TIG* tigecycline, *PIP* piperacillin, *PIP/TZB* piperacillin/tazobactam, *ATM* aztreonam, *LEV* levofloxacin, *iw* incubator water

### Antimicrobial susceptibility testing

Drug-resistance profiles were consistent between the 18 NDM-1-producing *K. pneumoniae* clinical isolates (M1–M18) and the one NDM-1-producing *K. pneumoniae* strain obtained from incubator water (M19). All the 19 strains were highly resistant to the tested carbapenems, including meropenem and imipenem. The MIC values for meropenem were in the range of 32–128 μg/mL and those of imipenem ranged from 4 to >128 μg/mL. Nineteen strains exhibited discrepant-level resistance to aztreonam, six isolates were sensitive, seven isolates were intermediate, and the rest six were resistant. The MIC values for the other tested β-lactam antibiotics were high (>128 μg/mL) in all tested strains. Tigecycline exhibited potent activity against all tested strains, none tigecycline reisitant strain was detected. All the isolates remained susceptible to ciprofloxacin and amikacin. These results are summarized in Table 1.

### PFGE and MLST typing

**Fig. 1:**
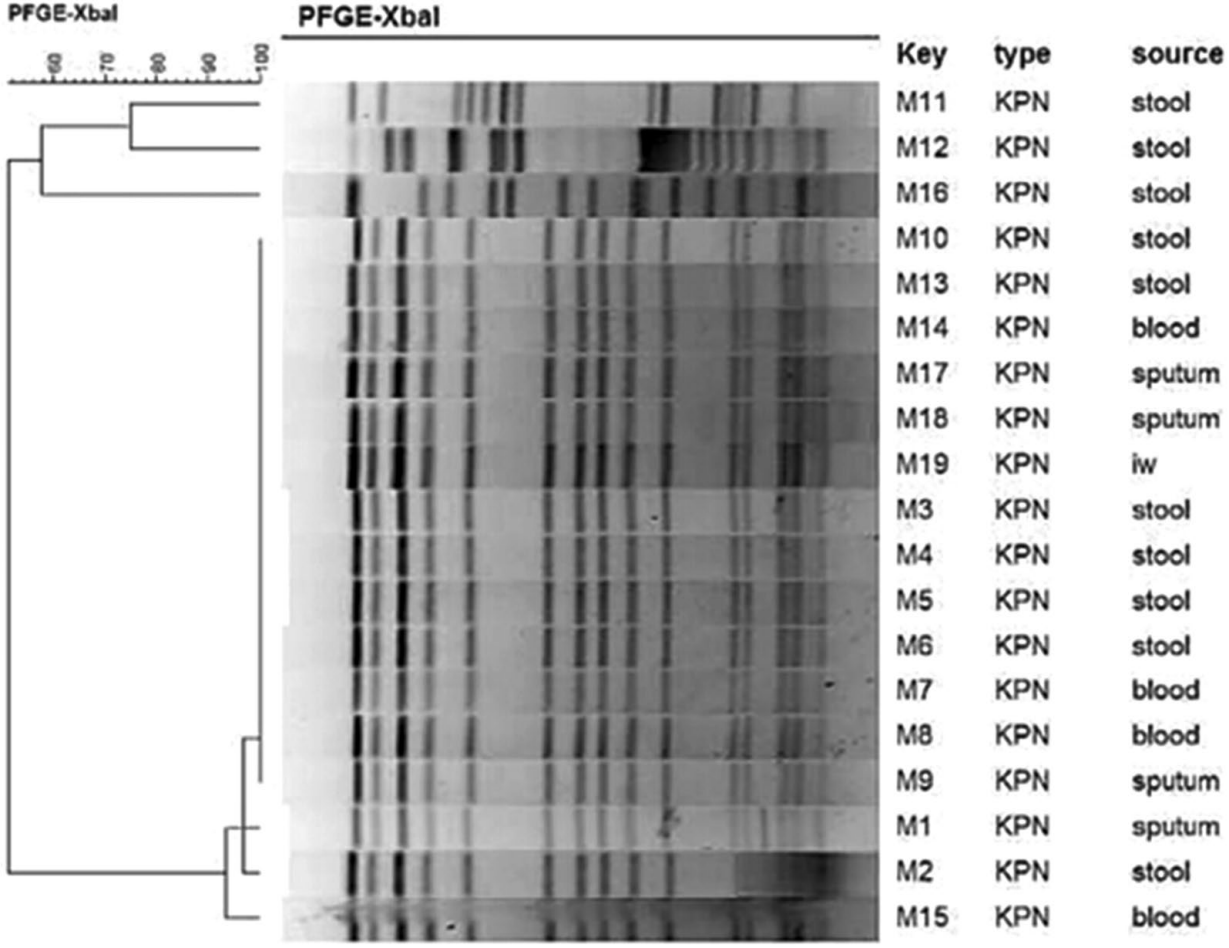
Dendrogram of PFGE patterns of chromosomal DNA restriction fragments from 19 *K. pneumoniae* isolates in present study. *KPN* *Klebsiella pneumoniae*, *iw* incubator water

PFGE patterns of the XbaI DNA digests of 19 *K. pneumoniae* isolates were obtained. Gel images were input into BioNumerics and phylogenetic tree was built for cluster analysis (Fig. 1). PFGE revealed four cluster among 19 *K. pneumoniae*. One cluster of 16 closely related isolates was found that exhibited >90 % similarities. MLST analysis showed that all the 19 *K. pneumoniae* strains identified here were defined as a single sequence type (ST105) with the allelic profile 2-3-2-1-1-4-18.

### Plasmid analysis and bacterial conjugation

Carbapenems resistance was successfully transferred from all *K. pneumoniae* isolates to *E. coli* J53 (Az^R^) by conjugation. The MIC values of the 19 transconjugants were tested, and all *E. coli* transconjugants exhibited significantly reduced carbapenem susceptibility to the tested carbapenems, including imipenem and meropenem, as compared to *E. coli* J53 (Az^R^). Meanwhile, the transconjugants were resistant to β-lactam antibiotics, although not to aztreonam, and were susceptible to quinolones and aminoglycosides (Table 2). Analysis of plasmids harbored by M1–M19 and transconjugants revealed the presence of two plasmids (ca. 50 and ca. 2.3 kb), while the transconjugants only acquired the ca. 50-kb plasmid. PCR analysis confirmed that the plasmid present in transconjugants harbored both *bla*_NDM-1_ and *bla*_SHV-1_. PCR-based inc/rep typing method showed that FIA, FIB, FIC, and F replicons were positive in all *bla*_NDM-1_- encoding plasmids, which belonged to IncFI incompatibility group.

**Table 2:** The results of antibiotic susceptibility testing of transconjugants in present study

Strains	MEM	IPM	CAZ	FOX	AMK	TIG	PIP	PIP/TZP	ATM	LEV
M1-J53	32	4	64	64	1	<0.125	>128	>128/4	0.5	<0.06
M2-J53	32	4	64	64	1	<0.125	>128	>128/4	0.5	<0.06
M3-J53	16	4	128	64	1	<0.125	>128	>128/4	0.5	<0.06
M4-J53	32	16	128	64	1	<0.125	>128	>128/4	0.5	<0.06
M5-J53	32	4	64	64	1	<0.125	>128	>128/4	0.5	<0.06
M6-J53	32	4	64	64	1	<0.125	>128	>128/4	0.5	<0.06
M7-J53	32	8	64	64	1	<0.125	>128	>128/4	0.5	<0.06
M8-J53	32	4	64	64	1	<0.125	>128	>128/4	0.5	<0.06
M9-J53	32	4	128	64	1	<0.125	>128	>128/4	0.5	<0.06
M10-J53	32	4	64	64	1	<0.125	>128	>128/4	0.5	<0.06
M11-J53	16	4	>128	64	1	<0.125	>128	>128/4	0.5	<0.06
M12-J53	32	8	64	64	1	<0.125	>128	>128/4	0.5	<0.06
M13-J53	32	4	64	64	1	<0.125	>128	>128/4	4	<0.06
M14-J53	32	4	64	64	1	<0.125	>128	>128/4	2	<0.06
M15-J53	32	8	64	64	1	<0.125	>128	>128/4	0.5	<0.06
M16-J53	32	4	64	64	1	<0.125	>128	>128/4	0.5	<0.06
M17-J53	32	4	32	64	1	<0.125	>128	>128/4	0.5	<0.06
M18-J53	32	16	64	64	1	<0.125	>128	>128/4	4	<0.06
M19-J53	32	4	64	64	1	<0.125	>128	>128/4	4	<0.06
*E. coli* J53	<0.125	<0.125	<0.125	8	1	<0.125	4	2/4	0.5	<0.06

*MEM* meropenem, *IPM* imipenem, *CAZ* ceftazidime, *FOX* cefoxitin, *AMK* amikacin, *TIG* tigecycline, *PIP* piperacillin, *PIP/TZB* piperacillin/tazobactam, *ATM* aztreonam, *LEV* levofloxacin

## Discussion

Emergence of NDM-1-producing *Enterobacteriaceae* have disseminated worldwide from the Indian subcontinent brought about problems regarding therapy and control. In China, plasmids encoding *bla*_NDM-1_ have been identified in *Enterobacteriaceae* isolates in several regions including Beijing, Shanghai, Hong Kong, and Shandong province, the size and Inc-type of the plasmids harbouring *bla*_NDM-1_ were vary from ~50 to ~336 kb including InX3, IncL/M, IncA/C, and IncN incompatibility group [11, 21–23]. In present study, the plasmid harbouring the *bla*_NDM-1_ belonged to the IncFI-type, which were different from previously replicon type reported in China before. Plasmid replicon types were related to the dissemination of resistance genes [22]. Due to its presence in all the 19 CREs in this study, this IncFI plasmid may be responsible for the dissemination of the *bla*_NDM-1_ in this area.

Moreover, dissemination of *bla*_NDM-1_ is associated with MLST type [24]. NDM-1-producing *K. pneumoniae* have been reported in different countries, and belonged to various kinds of MLST types, including ST11, ST14, ST17, ST25, ST147, ST149, ST231, ST340, and ST1043 [24–29]. Our datas indicated that all 19 NDM-1-producing *K. pneumoniae* strains belong to the same type, viz., ST105, which was different from previous types reported before. PFGE analysis showed 4 clusters for 19 ST105 strains. Among them, one cluster of 16 closely related isolates was found that exhibited ≥90 % similarities including the strains detected in incubator water. Thoese results suggested that 19 NDM-1-producing *K. pneumoniaee* strains were clonally related and easily spread to different patients in NICU ward, environmental reservoirs such as incubator water may contribute to the spread of these organisms within hospital. A previous research showed that *bla*_NDM-1_ gene had disseminated in the NICU via different Gram Negative Bacilli (*E. coli*, *A. baumannii*, *S. maltophilia* and/or *K. pneumoniae*) harbouring *bla*_NDM-1_ [30]. However, it is unclear how the *bla*_NDM-1_ was introduced into the NICU ward in Kunming City Maternal and Child health Hospital. We suspect that isolates in this study represent a novel ST and that autochthonous clones are locally acquiring plasmids carrying the *bla*_NDM-1_, as has been reported previously [27], more research would be needed to uncover it.

In addition, the average days for hospitalization in Kunming City Maternal and Child health Hospital is 6.06 days at present. However, the average hospital stay of the 17 patients including in this study was 18.9 days (more than trebled of average days of hospitalization in this hospital), prolonged hospitalization may contribute to spreading of the ST105 strains in NICU ward.

The *bla*_IMP-4_ carbapenemase-encoding genes have also been detected in the part of NDM-1-producing strains (7/19). *Klebsiella pneumoniae* strains co-harbouring *bla*_NDM-1_ and *bla*_IMP-4_ have been identified in China previously, when they were found to be colocalized on a ca. 300-kb plasmid [31]. Plasmid analysis in this study showed that transconjugants acquired a ca. 50-kb plasmid. We analyzed the genomic DNA and plasmid DNA of 19 transconjugants by PCR, and confirmed only the presence of the *bla*_NDM-1_ and *bla*_SHV-1_. This suggested that *bla*_IMP-4_ was not on the ca. 50-kb IncFI plasmid along with *bla*_NDM-1_. We speculate that the *bla*_IMP-4_ gene may lie on the chromosome or a high molecular weight non-conjugative plasmid of which was not detected by the methodology used, further research was needed to uncover it. *Bla*_CTX-M-15_ and *bla*_NDM-1_ have a common origin in the Indian subcontinent [25] and *bla*_CTX-M-15_ had been identified in the NDM-1-producing strains, irrespective of whether the genes were located on the same plasmid [32, 33] or not [34]. Our study showed the presence of *bla*_CTX-M-15_ along with *bla*_NDM-1_ in all strains, but none of the 17 patients or their family had any epidemiological link to the Indian subcontinent. AmpCs always been detected with NMD-1 producers [28], but *bla*_ACT-1_*, bla*_CMY_, and *bla*_DHA-1_ were not been found in this study. Although *qnrB4*, *qnrS1* and *aacA4* were detected in some strains, levofloxacin and amikacin maintained a good antibacterial activity in vitro.

Nineteen transconjugants showed increased MIC values for the tested carbapenems as compared with *E. coli* J53 (Az^R^). The MIC values of 19 transconjugants for meropenem and imipenem ranged from 16 to 32 μg/mL and 4 to 16 μg/mL respectively, which was more than fourfold higher than those of *E. coli* J53. Due to the transconjugants only acquired *bla*_NDM-1_ and *bla*_SHV-1_, we concluded that *bla*_NDM-1_ was primarily responsible for the high MIC values of carbapenems. In comprehensive consideration of both the MIC values of clinical isolates (M1–M18) and their transconjugants, *bla*_IMP-4_ and *bla*_CTX-M-15_ also played a role in conferring resistance to the carbapenems.

## Conclusions

Hospital environment such as incubator water may be the diffusion reservoirs of NDM-1-producing bacteria. Personal contact between the caregivers and the patients hospitalized in the same ward is the most likely transmission route. However, it can not determine how this clone was introduced into the hospital, ST105 *K. pneumoniae* may have been spreading in hospitals in the region and their prevalence may be increasing. Therefore, nosocomial surveillance system should play a more important role in the infection control to limit the spread of NDM-1-producing pathogens.

## Abbreviations

CLSI: Clinical and Laboratory Standards Institute
CPE: carbapenemase-producing *Enterobacteriaceae*
CRE: carbapenem-resistant *Enterobacteriaceae*
ESBL: extended-spectrum β-lactamase
MDR: multidrug-resistant
MLST: multilocus-sequence typing
NICU: neonatal intensive care unit
PFGE: pulsed field gel electrophoresis
